# Hyaluronic acid-coated Poly(L-lactide-co-1,3-trimethylene carbonate) modulate early cellular-scaffold interactions and osteogenic potential: a comprehensive *in vitro* and *in vivo* evaluation using mesenchymal stromal cells

**DOI:** 10.3389/fbioe.2026.1740154

**Published:** 2026-01-27

**Authors:** Øyvind Goksøyr, Mohammed A. Yassin, Tove Kivijärvi, Salwa Suliman, Annika Rosén, Anna Finne-Wistrand, Kamal Mustafa

**Affiliations:** 1 Centre of Translational Oral Research (TOR) – Tissue Engineering Group, Department of Clinical Dentistry, University of Bergen, Bergen, Norway; 2 Department of Oral and Maxillofacial Surgery, Haukeland University Hospital, Bergen, Norway; 3 Department of Fibre and Polymer Technology, KTH Royal Institute of Technology, Stockholm, Sweden

**Keywords:** biofabrication in dentistry, polymer scaffolds, bone tissue engineering, hyaluronic acid, mesenchymal stromal cells, surface modification

## Abstract

**Introduction:**

Synthetic polymers are widely used in scaffold fabrication but are generally bioinert and hydrophobic, limiting cell adhesion and interaction. Hyaluronic acid (HA) is a ubiquitously expressed polycarbohydrate and a key extracellular matrix component that regulates tissue hydration, cell adhesion, motility, and regeneration. Incorporating HA into synthetic materials presents a promising strategy to enhance hydrophilicity and support biological functions, such as cell adhesion and osteogenic differentiation. This study investigated the effects of HA coating on the physicochemical characteristics and osteogenic potential of poly(L-lactide-co-1,3-trimethylene carbonate) (PLATMC) scaffolds.

**Methods:**

PLATMC scaffolds were coated with HA via immersion. HA stability during sterilization and culture was assessed alongside release kinetics. BMSC responses and osteogenic differentiation were evaluated *in vitro* and *in vivo* over six months. Scaffold wettability was analyzed to determine changes in surface hydrophilicity following HA coating.

**Results:**

HA coating improved scaffold wettability in a concentration-dependent manner. HA release was characterized by burst kinetics, becoming undetectable after a few days under *in vitro* conditions, indicating that HA-driven effects are expected to be strongest during early cell–material interactions. *In vitro*, human BMSC showed RHAMM upregulation across all HA groups and CD44 upregulation in the 0.5% HA group after 24 hours. Rat BMSC exhibited increased osteocalcin expression, suggesting enhanced osteoinductive activity, corroborated by a trend toward osteopontin upregulation in human BMSC. *In vivo*, μCT analysis revealed higher tissue density surrounding HA-coated scaffolds at 8 weeks compared to 6 months in a rat subcutaneous implantation model.

**Conclusion:**

HA coating improved scaffold hydrophilicity and promoted early cell adhesion and osteogenic signaling. The findings indicate that HA-coated PLATMC scaffolds support early cellular engagement and osteoconductivity, while long-term outcomes are likely governed by intrinsic scaffold properties. These results highlight the potential of HA-coated PLATMC scaffolds for biofabrication in dentistry, particularly in oral and maxillofacial bone regeneration.

## Graphical Abstract

**Figure:**
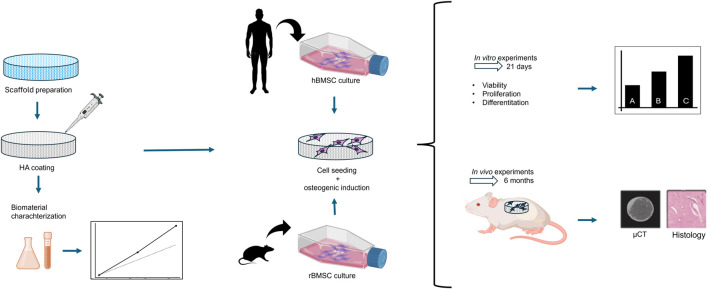

## Highlights

- Hyaluronic acid coating improved PLATMC scaffold wettability and BMSC adhesion
- HA was rapidly released from the scaffold under *in vitro* culture conditions
- HA-coated scaffolds upregulated RHAMM, CD44, and osteogenic markers in BMSC

## 1 Introduction

Bone is a highly vascularized connective tissue that protects internal body parts and provides mechanical support for skeletal locomotion. Despite its regenerative capacity, the repair of complex bone defects remains a significant clinical challenge. Bone tissue engineering (BTE) is acknowledged as a promising approach to the treatment and regeneration of bone defects, offering several advantages over conventional clinical therapies (Po et al., 2009). A key element in BTE is the development of a supportive matrix, typically comprising scaffolds, multipotent cells, signaling molecules, or their combinations (Langer and Vacanti, 1993; Murphy et al., 2013).

Among the components of this matrix, the three-dimensional scaffold plays a central role. It can be fabricated from a variety of natural or synthetic grafting materials. An ideal scaffold should be biocompatible, degradable, and highly porous, while also mimicking the structural and chemical composition of the native bone extracellular matrix (ECM) (Murphy et al., 2013). In addition to preserving the anatomical shape and structure of the defect (Arvidson et al., 2011), scaffolds can act as carriers for cells or signaling molecules (Seeherman and Wozney, 2005; Bayer, 2020) or serve as temporary ECM substitute (Nair and Laurencin, 2007; Amini and Nukavarapu, 2012), thereby supporting both endogenous and implanted cells.

Various materials have been investigated for scaffold fabrication in previous BTE studies. Synthetic polymers are particularly interesting due to the possibility for tailoring mechanical and degradation properties to suit different applications (Arvidson et al., 2011). One such material is poly(L-lactide-co-1,3-trimethylene carbonate) (PLATMC), synthesized through the copolymerization of trimethylene carbonate (TMC) and L-lactic acid. This copolymer exhibits enhanced flexibility, and its degradation products induce minimal local inflammation, attributes beneficial for biomedical and tissue engineering applications (Jain et al., 2020; Fuoco et al., 2019). Additionally, PLATMC has been shown to promote osteogenic differentiation in mesenchymal stromal cells (Neuss et al., 2011; Hassan et al., 2022). However, its inherent hydrophobicity (Fukushima, 2016; Bu et al., 2019; Thrivikraman et al., 2017) and lack of specific cell-binding motifs may ultimately result in poor cell adhesion (Arvidson et al., 2011; Nair and Laurencin, 2007).

Surface modification is widely explored to improve interactions between scaffolding materials and cells or their secreted proteins (Arvidson et al., 2011). Strategies such as coating (Zamboni et al., 2017), functional group incorporation (Tian et al., 2012), and plasma treatment (Yamada et al., 2021a) have been employed to enhance scaffold hydrophilicity and cytocompatibility. Bio-coating refers to the functionalization of three-dimensional synthetic scaffolds with biomolecules, such as proteins, peptides, growth factors, or antibodies, to induce targeted cellular responses and promote tissue regeneration (Lee et al., 2023). Immersion coating using aqueous media at neutral pH is considered a mild and effective technique, enabling scaffold modification without altering physicochemical properties or architecture (Nicodemus and Bryant, 2008; Joshy et al., 2019; Choi et al., 2021).

Functionalization of scaffolds with natural ECM components has shown promise in enhancing wettability and cell affinity (Bu et al., 2019). In particular, hyaluronic acid (HA) has received considerable attention for biomedical use (Zhai et al., 2020; Zha et al., 2016; Casale et al., 2016). HA is a glycosaminoglycan (GAG) and a major component of the ECM, contributing to its structural, rheological, physiological, and biological functions (Laurent, 1987; Chen and Abatangelo, 1999). Furthermore, HA is a hydrophilic, linear polysaccharide composed of repeating disaccharide units of α-1,4-D-glucuronic acid and β-1,3-N-acetyl-D-glucosamine, and it has been used as an injectable hydrogel in BTE applications (Zhu et al., 2017; Necas et al., 2008). However, the low mechanical strength of injectable HA limits its potential in load-bearing BTE contexts (Collins and Birkinshaw, 2013).

The biological activity of HA is strongly influenced by its molar mass (Xing F. et al., 2020; Bohaumilitzky et al., 2017). Although high molar mass HA has been associated with increased expression of osteogenic markers (Zhao et al., 2015), the precise effects of HA molar mass and concentration remain unclear. HA also contributes to cell proliferation and migration, wound healing, and the regulation of extracellular water homeostasis (Solis et al., 2012; Dicker et al., 2014; Dickinson and Gerecht, 2016). Its facilitation of cell migration may be attributed to both its ability to form a hydrated matrix and its interactions with cell surface receptors, such as RHAMM, which mediate directional migration (Chen and Abatangelo, 1999). Another major HA-specific ligand, CD44, is involved in regulating cell proliferation and adhesion (Aruffo et al., 1990; Knopf-Marques et al., 2016). When bound to membrane receptors, HA forms a protective and selective coating around the cell surface (Campoccia et al., 1998). It has also been reported to enhance bone regeneration and is a component of the early fracture callus (Unnithan et al., 2017).

The primary aim of this study was to evaluate whether surface modification of PLATMC scaffolds with HA could enhance cellular behavior and osteogenic differentiation, ultimately supporting ectopic bone formation. The study examined the effect of three HA concentrations on PLATMC scaffold wettability. Attachment, proliferation, and differentiation of bone marrow-derived mesenchymal stromal cells (BMSC) were assessed. In view of HA’s potential in BTE, an additional objective was to determine the impact of sterilization and coating procedures on the integrity of HA. Importantly, to assess translational relevance, *in vivo* evaluations were conducted, as *in vitro* models often fail to accurately predict performance in preclinical animal studies, which are essential for clinical application (Hatt et al., 2023). To further assess osteogenic capacity, ectopic (*de novo*) bone formation was evaluated in a subcutaneous implantation model using osteogenically committed rat-derived BMSC (rBMSC). This ectopic site was chosen to investigate the intrinsic osteoinductive properties of the scaffolds independent of native bone cues. Evaluations were conducted over a period of up to 6 months to capture both early and long-term tissue responses.

In addition to its relevance for bone tissue engineering, this work contributes to Regenerative Dentistry by proposing a surface modification strategy that may be translated to oral and maxillofacial applications. Scaffold-based approaches are central to dental and craniofacial bone regeneration (Dissanayaka and Zhang, 2020), and enhancing scaffold bioactivity through HA-coating could improve outcomes in alveolar ridge augmentation, periodontal regeneration, and implantology, and the repair of larger craniofacial defects.

## 2 Materials and methods

### 2.1 Scaffolding and coating materials

Poly(L-lactide-co-1,3-trimethylene carbonate) (Resomer® LT706S) was sourced from Evonik Industries. The copolymer’s composition, number-average molar mass, and dispersity were analyzed prior to scaffold fabrication using proton nuclear magnetic resonance (^1^H NMR) and size-exclusion chromatography (SEC) in chloroform (60 mol% LLA and 40 mol% TMC; Mn = 146 kDa; Ð = 1.5). Hyaluronic acid sodium salt derived from *Streptococcus equi* (a bacterial glycosaminoglycan polysaccharide; Mn 1.5–1.8 MDa) was obtained from Merck/Sigma-Aldrich®.

### 2.2 Size exclusion chromatography (SEC)

The number average molar mass (*M*_*n*_) weight average molar mass (*M*_*w*_) and dispersity (*Ð*) of hyaluronic acid were characterized using a Dionex Ultimate-3000 HPLC system (Dionex, Sunnyvale, CA, USA). Hyaluronic acid was solubilized in MQ-H2O (up to 2 mg mL^-1^) and filtered through 0.2 µm nylon filters prior to injection (40 µL per injection). Three serial-coupled columns (dimension 300 × 8 mm, particle size 10 µm) with pore sizes of 3 nm, followed by 2 columns of 100 nm, were used at 40 °C. Sodium hydroxide (100 mM) was used as an eluent, at a flow rate of 1 mL/min^-1^. A Waters refractive index was used as a detector (Waters-410, Milford, MA, USA) and pullulan polymers were used as standards (*M*_*n*_ range 342–708,000 Da). Chromeleon 7.1 was used to process data.

### 2.3 Scaffold fabrication and sterilization

The scaffolds were fabricated by conventional salt-particulate leaching techniques described previously (Mikos et al., 1994), with some modifications. Poly(L-lactide-co-1,3-trimethylene carbonate) was dissolved in chloroform (1 g 10 mL^-1^) and mixed with sieved sodium chloride particles (particle size range 75–500 µm), then cast into a glass dish to a thickness of 1.5 mm. The solvent was allowed to evaporate slowly and disc-shaped scaffolds 10 mm in diameter were punched out. The salt particles were leached out over 7 days in double-distilled water and then dried prior to use. The scaffolds were sterilized by treatment with 70% ethanol twice, washed three times with PBS, and finally irradiated with ultraviolet light (UVC: 254 nm–4.9 W) for 2 h.

### 2.4 Hyaluronic acid preparation and scaffold coating

Hyaluronic acid sodium salt was sterilized by autoclaving at 121 °C for 20 min. The HA powder was subsequently dissolved in Type 1 ultrapure H_2_O by agitation at 4 °C overnight. The selected HA concentrations (0.1%, 0.25%, and 0.5% w/v) were determined based on the physicochemical properties of the HA used, as 0.5% represents the maximum solubility at the given molar mass under the applied conditions, while lower concentrations were included to enable comparative evaluation across a broader concentration range.

For coating, the scaffolds were submerged overnight in HA, in 48-well culture plates (NUNC™, Thermo Fisher Scientific, Waltham, MA, USA), on a shaker at 4 °C. The scaffolds were then transferred to empty wells and frozen before water was extracted in a freeze dryer (FreeZone 2.5, LABCONCO, Kansas City, Missouri, USA). The culture plates with coated and uncoated control scaffolds were then sealed in plastic bags and stored at −80 °C until use.

### 2.5 Assessing surface wettability

When a droplet is placed on a surface, its shape and contact angle are dependent on the ratio of adhesion between substrate and droplet, and the forces of cohesion within the droplet (Vuckovac et al., 2019; Law, 2014). The surface wettability of the scaffolds was evaluated by a modified contact angle assay: Fifty millilitres of Dulbecco’s modified eagle culture medium (DMEM) (Invitrogen, Carlsbad, CA, USA) were loaded onto all the scaffolds in the experimental and control groups and lateral-view pictures were taken. Mean contact angle approximations from both sides of the droplet were made using ImageJ software.

### 2.6 Scaffold characterization using microcomputed tomography (µCT)

After sterilization and coating, the scaffolds were characterized by µCT, using a Skyscan 1172 x-ray µCT imaging system (Bruker, Kontich, Belgium) as previously described, with some modifications. Briefly, the x-ray source was operated at 50 kV and 200 μA, without a filter. 2-dimensional CT images were captured every 0.6° through 180° rotation, then reconstructed by Skyscan NRecon software at thresholds of 57–255. Regions of interest were selected, and three-dimensional analysis was performed using Skyscan CTAn software (Sharma et al., 2018), and the following characteristics were determined for all scaffold groups: Total porosity, surface area, mean pore diameter and fractal dimension.

### 2.7 Stem cell culture

#### 2.7.1 Ethical statement for obtaining and utilizing human mesenchymal stromal cells (BMSC)

BMSC were isolated from human tissue samples as previously described (Mohamed-Ahmed et al., 2018). The samples were obtained with informed consent from patients who underwent routine surgery at Haukeland University Hospital, Bergen, Norway. This study received ethical approval from Regional Committees for Medical and Health Research Ethics (REK) in Norway; (2013-1248/REK sør-øst C) (Mohamed-Ahmed et al., 2018). All procedures and methods were performed in accordance with their regulations.

### 2.8 *In vitro* cell expansion

BMSC were expanded in DMEM culture medium (Invitrogen, Carlsbad, CA, USA) supplemented with 10% fetal bovine serum (FBS) (Hyclone–GE Healthcare Life Sciences, South Logan, UT, USA) and 1% penicillin/streptomycin (GE Healthcare Life Sciences. The medium was changed every 3–4 days. Cells were subcultured at 90% confluence and characterized at passages 3 and 4 by immunophenotyping and trilineage differentiation, as previously described (Mohamed-Ahmed et al., 2018; Dominici et al., 2006).

### 2.9 Biological evaluation of scaffolds *in vitro*

Three experimental groups, with HA coating concentrations of 0.1%, 0.25% and 0.5% respectively, were compared with the uncoated control group. For all *in vitro* experiments, cells were seeded onto scaffolds and cultured for up to 21 days. Briefly, 2 × 10^5^ human BMSC from passage 5 were seeded using 50 µL aliquots on the scaffolds. The scaffolds were then incubated at 37 °C 5% CO_2_ for 60 min before the addition of 450 µL medium to each well (48-well plates (NUNC™, Thermo Fisher Scientific, Waltham, MA, USA). After incubating overnight, the expansion medium (DMEM) was changed to a medium with osteogenic supplements (10 nM dexamethasone, 0.5 mM ascorbic acid, and 7 µM β-glycerophosphate) then cultured for up to 21 days. The medium was changed twice a week.

### 2.10 Seeding efficiency

Seeding efficiency was calculated by seeding 2 × 10^5^ cells on dry scaffolds and in empty wells as controls. After 4 h the scaffolds were removed, and the cells remaining in the wells and supernatant medium were counted Countess II™ (Invitrogen).

Calculations were made according to the following equation:

$$\text{Seeding efficiency}=\frac{\left(\text{Initial}\;\text{seeding}\;\text{number}-\text{cells}\;\text{remaining}\;\text{in}\;\text{wells}\right)}{\text{Initial}\;\text{seeding}\;\text{number}}\times 100$$

### 2.11 Hyaluronic acid release and degradation *in vitro*

Scaffolds were seeded with cells suspended in medium without phenol red. Medium was collected for analysis of HA content at 0, 1, 6 and 18 h after seeding. At every media change, samples from old and newly added medium were collected and stored in −80 °C until turbidity assay, as previously described (Oueslati et al., 2014), with some modifications: 0.1M phosphate buffer was used as diluent and 2% NaOH as blank. The reagent was prepared by dissolving 2.5 g cetyltrimethylammonium bromide (CTAB) (Sigma-Aldrich) in 100 mL 2% NaOH. All reagents were brought to 37 °C and equal amounts (50 µL) of diluent and sample were loaded in a 96-well plate and incubated for 15 min at 37 °C, before adding 100 µL CTAB reagent (1:1 with the diluted samples) and vortexed for 10 s. The plates were incubated for 10 min at 37 °C before absorbance was read at 600 nm.

### 2.12 Cell attachment

#### 2.12.1 Scanning electron microscopy (SEM)

Scanning electron microscopy was used to visualize surface topography and cell attachment. At 3 and 14 days, seeded scaffolds were washed and fixed before dehydration in alcohol. The samples were mounted on 12 mm aluminum studs, sputter coated with a 10 nm layer of Pd/Au (Jeol JFC-2300HR high resolution fine coater (Tokyo, Japan)) and imaged (Jeol JSM-7400F (Tokyo, Japan)).

#### 2.12.2 Confocal microscope imaging

After 24 h and 7 days, samples were collected, fixed, and prepared for imaging. In short, medium was discarded, and the samples were washed with PBS and fixed in 4% paraformaldehyde (PFA) for 10 min. The samples were then washed for 2 × 10 min in 0.1% triton X in PBS (PBST). Blocking was conducted using 0.1% PBST supplemented with 10% normal goat serum for 60 min. The scaffolds were then incubated at 4 °C overnight in primary antibody mouse-anti-human N-cadherin 1:500. After 24 h, scaffolds were washed in 0.1% PBST and incubated with secondary antibodies Alexa 546 goat-anti-mouse 1:200, Alexa 488 Phalloidin 1:200 and DAPI 1:2500. Scaffolds were imaged using a Leica TCS SP8 STED 3×. Images were constructed using ImageJ software (stack: 30, z-step 0.85 µm).

### 2.13 Assessing cell viability by LIVE/DEAD staining

Cell viability was evaluated by live/dead (Invitrogen, Thermo Fischer scientific) staining, in accordance with the manufacturer’s instructions. Briefly, at day 3 and day 21, scaffolds were collected and washed twice in PBS. Immunofluorescent dyes were prepared by diluting 2 mM Etidium homodimer-1 (stains dead cells red) and 4 mM Calsein-AM (stains live cells green) in PBS. The samples were then imaged using a light microscope (Olympus, Tokyo, Japan).

### 2.14 DNA quantification (PicoGreen™ assay)

To evaluate cell expansion, a Quant-iT™ PicoGreen™ dsDNA Assay Kit (Invitrogen) was used Briefly, scaffolds seeded with cells were washed thoroughly with PBS to remove residual culture medium and extracellular DNA, and subsequently fixed using 10% formalin, before incubation in 200 µL 0.02% SDS solution 0.02% proteinase K overnight. Picogreen dye was diluted 1:200 in 1× TE buffer and mixed with lysate 1:1. Fluorescence was read using a FluoSTAR plate reader at 485 nm excitation and 520 nm emission at 1, 7 and 14 days after osteogenic induction (n = 5). DNA quantification was used as an estimate of scaffold-associated cell number over time and does not provide a direct measure of cell proliferation rate, as it reflects the net balance between cell division, survival, and potential cell loss.

### 2.15 Quantitative polymerase chain reaction (RT-qPCR)

Total RNA was extracted from the cells using (Maxwell®, Promega, Madison, WI, USA) following the manufacturer’s protocol. RNA concentrations were measured by spectrophotometry (ND-1000 Spectrophotometer, Nanodrop Technologies, Wilmington, DE, USA) and normalized to 300 ng for all samples. Following the manufacturer’s protocol, cDNA transcription was obtained by a Reverse Transcription kit (Applied Biosystems, Foster City, CA, USA) using a thermal cycler system (SimpliAmp, Applied Biosystems, Ca, USA). Subsequently, a real-time polymerase chain reaction was performed using TaqMan Fast Universal PCR Master MIX (Applied Biosystems). Markers for osteogenic differentiation were assessed at 7, 14 and 21 (*n* = 5) days, and surface markers were evaluated after 24 h and 5 days. The Genes assessed are summarized in Table 1.

**TABLE 1:** Real time PCR primers.

Gene	Abbreviation	Function	Assay ID	Amplicon length
HA specific genes				
Cluster of differentiation 168	RHAMM/CD168	Receptor for hyaluronic acid mediated motility	Hs00234864_m1	98
Cluster of differentiation 44	CD44	Cell surface adhesion molecule	Hs99999195_m1	61
Osteogenesis-related genes				
Runt related transcription factor	RUNX2	Marker for osteoblast differentiation	Hs01047973_m1	86
Collagen 1	COL1	Marker for component of ECM	Hs00164099_m1	68
Osteopontin (Bone sialoprotein 1)	SPP1	Osteoblast marker (organic component of bone).	Hs00959010_m1	84
Osteocalcin (Bone gamma-carboxyglutamate protein)	BGLAP	Osteoblast-specific marker	Hs01587814_g1	138
Housekeeping gene				
Glyceraldehyde-3-phosphate dehydrogenase	GAPDH	Marker for cell metabolism	Hs02758991_g1	93

### 2.16 Evaluation of mineralized deposits *in vitro*

Mineralized deposits on the scaffolds were assessed after 21 days in culture. The scaffolds were washed in PBS and covered with 2% Alizarin red S staining solution (Sigma-Aldrich), before incubation in the dark for 45 min at room temperature. Macrographs were captured using a light microscope. (Leica M205 C, Leica Microsystems GmbH, Wetzlar, Germany). The staining was dissolved using 100 mM cetylpyridinium chloride (Sigma-Aldrich) under agitation for 4 h before reading absorbance at 540 nm (FLUOstar OPTIMA Microplate Reader; BMG Labtech, Offenburg, Germany).

### 2.17 Animal experiments

#### 2.17.1 Approval for utilizing animal models and rBMSC

This study was approved by the Norwegian Animal Research Authority and conducted in accordance with the European Convention for the Protection of Vertebrates Used for Scientific Purposes (FOTS ID: 17734). In compliance with the ARRIVE guidelines (Kilkenny et al., 2010), a four-split back design was employed, allowing the implantation of four scaffolds per animal. This approach minimized the total number of animals required for the experiments.

#### 2.17.2 rBMSC isolation and charachterization

Human BMSC were used exclusively for the *in vitro* experiments, whereas rat BMSC (rBMSC) were employed only for the *in vivo* animal studies; supplementary analyses of rBMSC were performed solely to characterize the cell population used for implantation (Supplementary Material).

For the animal model, rBMSC were isolated from the femurs of male Lewis rats and maintained using a modified version of a previously established protocol (Song et al., 2014). Rat BMSC were used for implantation to match species-specific host responses. The cells were cultured in flasks containing minimum essential medium (αMEM; Invitrogen™, Carlsbad, California, USA) supplemented with 1% penicillin-streptomycin (PS) and 10% fetal bovine serum (FBS). At 90% confluence, the cells were passaged and sub-cultured. Characterization was performed at passages 3 and 4 through immunophenotyping and trilineage differentiation, as previously described (Yamada et al., 2021b).

#### 2.17.3 Cell adhesion and osteocalcin expression *in vitro* of rBMSC

rBMSC were seeded and cultured for 6 h and 7 days to evaluate initial and late cellular responses. Samples were prepared for confocal imaging as reported above, using the following antibodies and dilutions: DAPI 1:2500 (Invitrogen 62247), Phalloidin Alexa488 1:500 (Invitrogen A12379), Anti-alpha tubulin antibody 1:250 (Invitrogen 62204), Anti-osteocalsin antibody 1:250 (Novus Biologicals MAB1419), Anti-mouse Alexa 635 antibody 1:500 (A-31575 Invitrogen).

Additionally, rBMSC expansion and differentiation were evaluated *in vitro via* DNA content assessment on day 7 and mineralization on day 21, as reported above (Supplementary Material).

#### 2.17.4 Surgical procedures

Based on the *in vitro* results, 0.5% HA-PLATMC scaffolds were preselected for further investigation. The effect of HA coating on *in vivo* bone formation was evaluated using a subcutaneous implantation model in rats. Scaffolds were pre-cultured in osteogenically supplemented αMEM for 1 week prior to implantation.

Male Lewis rats (∼180 g, 6 weeks old; Naiser AS, Norway) were used in compliance with the Norwegian Animal Research Authority and the European Convention for the Protection of Vertebrates Used for Scientific Purposes. Animals were anesthetized with SevoFlo^®^ (sevoflurane; Abbott Laboratories, UK). Anesthesia was induced with 7% sevoflurane in a separate induction chamber, after which animals were positioned prone and anesthesia was maintained at 3% sevoflurane *via* a face mask. The dorsal area was shaved and disinfected with 5 mg/mL chlorhexidine ethanol.

Two small incisions were made along the vertebral column, and bilateral subcutaneous pockets were created using blunt dissection. One scaffold or scaffold-cell construct was implanted into each pocket, and the incisions were closed with Vicryl™ PLUS 4-0 sutures (Ethicon, Somerville, NJ, USA). Postoperatively, all animals received an intramuscular dose of buprenorphine (Temgesic^®^, 0.1 mg/kg) as an analgesic and were monitored daily for surgical wound status, food intake, activity, and signs of infection or discomfort.

At predetermined time points (2 and 6 months), the animals were euthanized by CO_2_ overdose with 30% CO_2_ volume displacement per minutefollowed by cervical dislocation. The scaffolds were harvested and stored in RNAlater® (Thermo Fisher Scientific, Waltham, MA, USA) at −80 °C for further analysis.

#### 2.17.5 Evaluation and quantification of ectopic bone formation

The harvested samples were subjected to radiographic analysis using a μCT scanner (Skyscan 1172VR) with an X-ray source set at 50 kV/200 μA and a 0.5 mm aluminum (Al) filter, achieving a resolution of 10 microns. Global thresholding was performed with a threshold level of 100/255. Bone volume (BV) and bone volume-to-tissue volume ratio (BV/TV) were quantified using CT-Ana software (Skyscan, Belgium). Three-dimensional surface rendering images were generated using the imaging software CTVoxVR.

#### 2.17.6 Histology

Scaffolds were dehydrated through a graded series of alcohol concentrations (70%–100%), cleared with Tissue Clear®, and embedded in paraffin wax. Sections (∼5 µm thick) were cut along the scaffold centerline, stained with hematoxylin and eosin (H&E), and subsequently scanned for analysis.

The automated processing protocol consisted of formalin fixation (2 × 1.5 h), dehydration with 70% ethanol (0.5 h), 96% ethanol (1 h), and 100% ethanol (4 × 1 h), followed by clearing with Tissue Clear® (2 × 1.5 h) and embedding in paraffin wax (4 × 1 h).

### 2.18 Statistical analysis

Quantitative data were analyzed using linear models, with post-estimation for multiple comparisons when appropriate. For RT-qPCR, ΔCT values were included directly in the regression model and visualized in the corresponding figures. A significance threshold of 5% was applied, and data presented as mean ± standard deviation (SD). Statistical analyses were performed in STATA (version 16; StataCorp, College Station, TX, USA). Graphs were generated using GraphPad Prism (version 7.04; GraphPad Software, San Diego, CA, USA).

## 3 Results

### 3.1 Hyaluronic acid coating significantly decreased surface contact angle in a concentration-dependent manner

**FIGURE 1:**
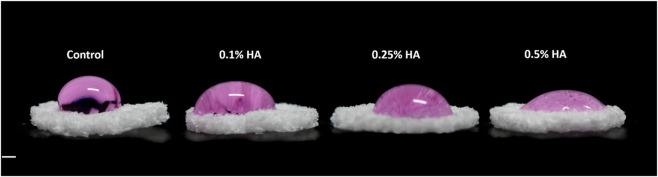
Surface wettability. Lateral view images of cell suspension 1 h after seeding. Scale bar = 1 mm.

Cell expansion medium was loaded onto modified and unmodified scaffolds in aliquots of 50 µL resembling cell seeding during experiments. The droplet was left undisturbed for 1 h before images were taken, coinciding with the time allowed for initial cell attachment to scaffold surfaces in our experiments (Figure 1). This demonstrates that the increase in HA concentration leads to a decrease in surface contact angle, in effect suggesting an increase in surface energy (Wenzel, 1936). Improved wettability is particularly advantageous for dental scaffold applications, as it can facilitate cell adhesion and proliferation. Quantitative analysis revealed a significant reduction in contact angle for HA-coated scaffolds compared with the unmodified (94° ± 7.2). The mean contact angle decreased to 79.5° ± 6.8 for 0.1% HA (*p* = 0.009), 68.5° ± 5.7 for 0.25% HA (*p* = 0.009), and 41.5° ± 5.9 for 0.5% HA (*p* < 0.001) (mean ± SD). These findings confirm a clear concentration-dependent improvement in surface wettability following HA coating.

### 3.2 The coating did not change the physical properties of the scaffolds as measured by µCT

The physical properties of the scaffolds were assessed by µCT before and after HA coating and are summarized in Table 2 and. Reconstruction of µCT images revealed porosities ranging from 84% to 89%. 3.3 Residual HA was detectable in the culture medium up to 4 days *in vitro*.

**TABLE 2:** CT characterization of physical properties of scaffolds.

Coating concentration (%)	Total porosity (%)		Surface area (mm^2^)		Mean pore diameter (mm)		Fractal dimension	
​	Mean	±sd	Mean	±sd	Mean	±sd	Mean	±sd
0.1	89.09	3.38	614.39	160.92	0.39	0.02	2.58	0.09
0.25	88.29	3.57	669.61	158.00	0.37	0.01	2.61	0.07
0.5	89.07	1.80	648.49	56.14	0.38	0.02	2.61	0.03
Control	84.58	3.92	730.83	170.07	0.38	0.02	2.64	0.06

Hyaluronic acid release into medium was measured by a turbidity assay as previously described (Oueslati et al., 2014). On adding medium to the sample there was an initial burst release of HA for the first 6 h, then a decrease in release with medium change, suggesting that most of the coating material was washed off during the first day in culture. Using the absorbance reading for the uncoated control group disclosed that residual HA could be detected in the medium after 4 days, equivalent to two media changes in both 0.25% and 0.5% HA groups, suggesting a dilution of the released HA with the media changes. The residual HA was highest in the high concentration (0.5% HA) group at all timepoints (*p* < 0.05), becoming undetectable after the second media change at 4 days in culture. The rapid release profile indicates the need for strategies to enhance HA retention, particularly for clinical applications such as alveolar ridge augmentation and periodontal regeneration.

### 3.3 The sterilization process led to significant degradation of HA, but HA did not degrade further *in vitro*

**FIGURE 2:**
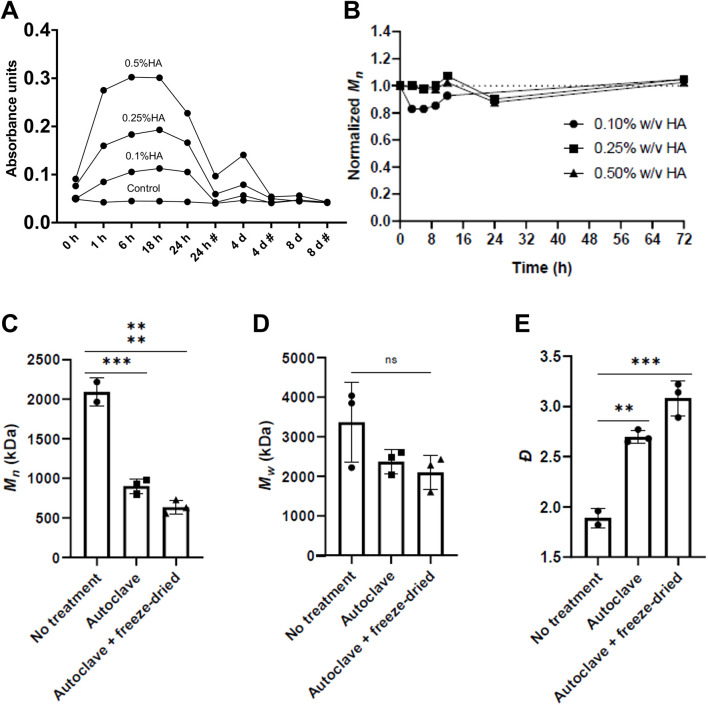
HA release and degradation. **(A)** Residual HA in culture medium. # Represents change of medium. **(B)** HA degradation *in vitro*. (n = 5). Data are presented as means ± SD. **(C–E)** Degradation impact on HA during pre-treatment illustrated a significant difference in Mn and Đ of HA after sterilization and freeze-drying compared to before (no treatment). Based on these data, it should be noted that the scaffolds coated with HA had an Mn corresponding to approximately half of the initial Mn. Determined from DionexUltimate-3000 HPLC system referenced to pullulan standards. N.S., not significant, *p ≤ 0.05, **p ≤ 0.01, ***p ≤ 0.001, ****p ≤ 0.0001 (*n* = 3; mean ± SD).

Before coating the scaffolds, HA was sterilized by autoclaving. This process significantly affected the number average molar mass (*M*_*n*_) of HA (p < 0.01). A similar reduction, although not significant, was observed for the weight average molar mass (*M*_*w*_), The freeze-drying process also led to a non-significant drop in molar mass. Following *in vitro* culture of scaffolds for up to 72 h, no further decrease in *M*_*n*_ was recorded (Figure 2).

### 3.4 Seeding efficiency was consistent across the scaffold groups

**FIGURE 3:**
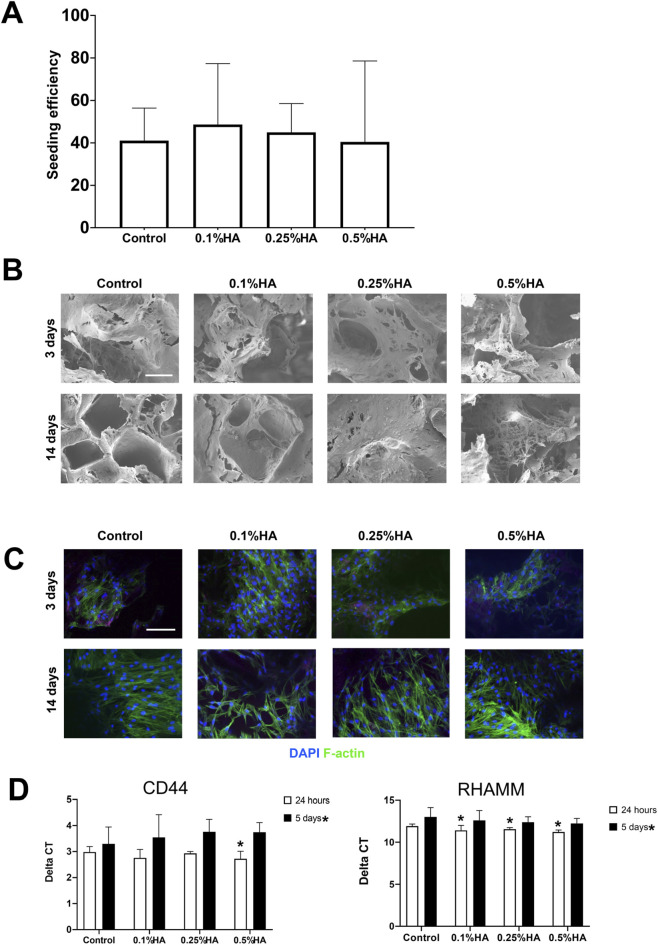
Evaluation of seeding efficiency and cell attachment to scaffolds. **(A)** Cell seeding efficiency. Data are presented as mean ± SD (*n* = 5). **(B)** Scanning electron microscopy and **(C)** immunofluorescence images of cell-seeded scaffolds after 3 and 14 days. Scale bar = 100 µm. **(D)** mRNA expression of HA specific surface markers on days 1 and 5 after seeding. Relative mRNA levels were normalized to GAPDH. Values are presented as mean ± SD of ΔCT. *p < 0.05 (*n* = 6).

After seeding, scaffolds were removed and the cells remaining in the wells were counted. No statistically significant inter-group differences were observed for cell seeding efficiency, which was generally in the range of 40%–48% (Figure 3A).

### 3.5 Cells morphology and attachment to the scaffolds

#### 3.5.1 SEM and confocal imaging

All scaffolds exhibited favorable cell attachment after 3 days. There were no visible intergroup differences in cell morphology: the cells appear flattened and well spread, bridging gaps on the scaffold surface.

Cell attachment was also confirmed using confocal imaging. Actin-filaments appeared more organized on day 14 than on day 3, with cells exhibiting similar morphology and distribution in all samples (Figures 3B,C).

### 3.6 HA-spesific receptors expression at gene level

Expression of surface-markers CD44 and RHAMM was significantly higher after 24 h than after 5 days. There was an overall trend for upregulation of the coated groups for CD44, significant for 0.5% HA, while expression of RHAMM was significantly higher for all coated groups after 24 h. On day 5 the HA coated groups had non-significant lower expression of CD44 and higher expression of RHAMM than the control group (Figure 3D).

These receptor-mediated responses emphasize the bioactive influence of HA functionalization, suggesting that HA-modified scaffolds may promote favorable early cellular interactions crucial for dental tissue engineering applications.

### 3.7 DNA quantification

**FIGURE 4:**
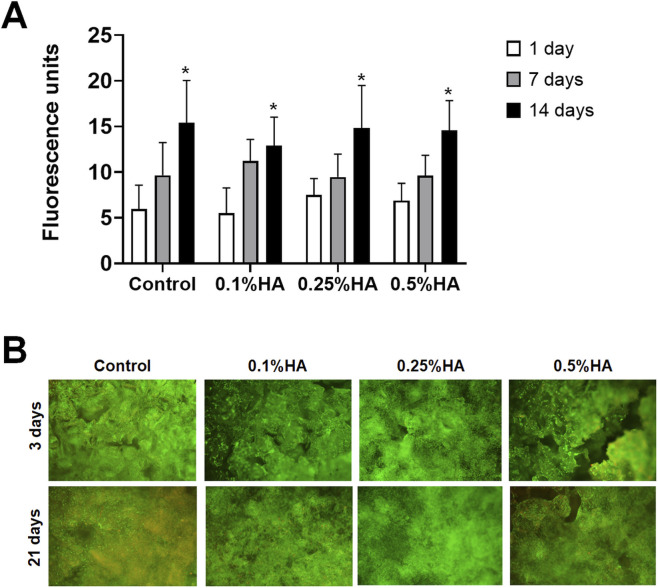
DNA content and cell viability. **(A)** DNA quantification after 1,7 and 14 days. Data are presented as mean ± SD. *p < 0.05 (*n* = 5). **(B)** Fluorescent images of seeded scaffolds after 3 and 21 days. Live cells are stained green and dead cells are stained red.

Total DNA was quantified at 1-, 7- and 14 days of culture. It should be noted that changes in DNA content reflect variations in total cell number rather than a direct proliferation rate, and therefore represent the combined outcome of cell expansion and cell survival over time. After 24 h, DNA content was similar in all groups. Incubation time showed an overall positive effect on DNA content, with a significant increase from day 7 to day 14 (p < 0.001) (Figure 4A)**.**

### 3.8 Cell viability

As depicted in Figure 4B, viability assay of the seeded cells shows an abundancy of live cells, with only a few dead cells visible on all scaffolds on both day 3 and day 21.

### 3.9 Osteogenic differentiation

The expression of collagen 1 (COL1) in BMSC was significantly downregulated over time in all HA groups. The expression of Runt-related transcription factor (RUNX2) was constant, suggesting a continuous differentiation rate. At 21 days, the expression of osteopontin (SPP1), a late marker of osteogenic differentiation, exhibited an upward trend in the 0.5% HA group, approaching statistical significance (p = 0.067). Osteocalcin (BGLAP) did not demonstrate notable changes in expression at any of the tested time points.

**FIGURE 5:**
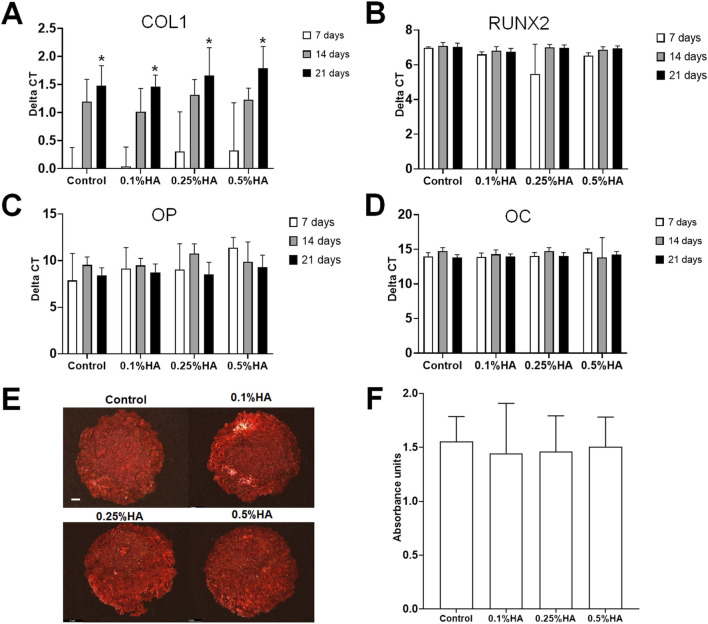
Evaluation of osteogenic differentiation. **(A–D)** mRNA expression of osteogenic differentiation markers on day 7, 14 and 21 after seeding. Relative mRNA levels were normalized to GAPDH. Values are presented as mean ± SD of ΔCT. *p < 0.05 (*n* = 5). **(E)** Macroscopic images of coated and uncoated scaffolds stained with Alizarin red S. Scale bar = 1 mm. **(F)** Quantification of Alizarin red S staining. Values are presented as mean ± SD (*n* = 4).

Positive alizarin red staining, indicating calcium deposition, was observed in all scaffolds. Relative quantification of the staining showed comparable results in all groups (Figure 5).

### 3.10 Animal experiments

#### 3.10.1 rBMSC: charachterization, cell adhesion, viability and osteogenic differentiation *in vitro*

As previously described (Yamada et al., 2021b), the cell batch used in this study was verified for phenotype, surface markers, and tri-lineage differentiation potential. No additional characterization was performed here.

On the surface of the control group, fluorescence imaging revealed cells maintaining a round morphology, similar to the cells on the HA coated surface. In addition, more cells were observed on the coated than the control scaffolds, which could suggest increased survival of the cells upon seeding. Further, after 7 days in culture, osteocalcin expression was evident on the HA-coated scaffolds, whereas it was absent in the uncoated control group (Supplementary Data).

#### 3.10.2 *de novo* mineralized tissue formation

**FIGURE 6:**
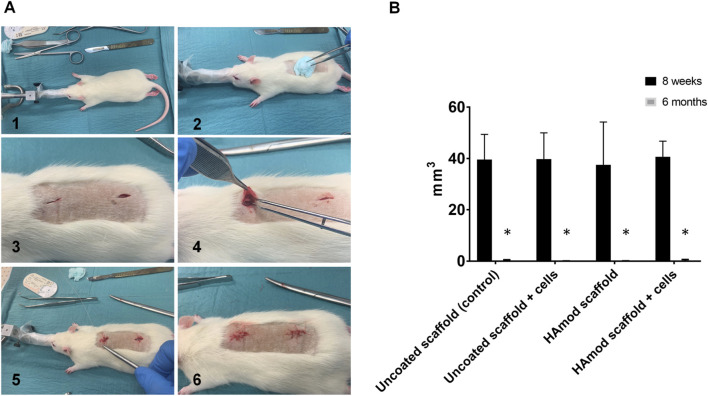
Surgical procedures and *in vivo* mineralization. **(A)** The rats’ heads were stabilized on a mask providing continuous flow of gas achieving surgical anesthesia. The surgical site was prepared, and incisions along the midline of the dorsum were made, creating pockets by blunt dissection. Scaffolds were placed before closure. **(B)** At 8 weeks, µCT showed significantly denser tissues, compatible with matrix secretion preceding osteoid deposition or the presence of pre-mineralized matrix, across all groups. By 6 months, a reduction in tissue density was observed.

**FIGURE 7:**
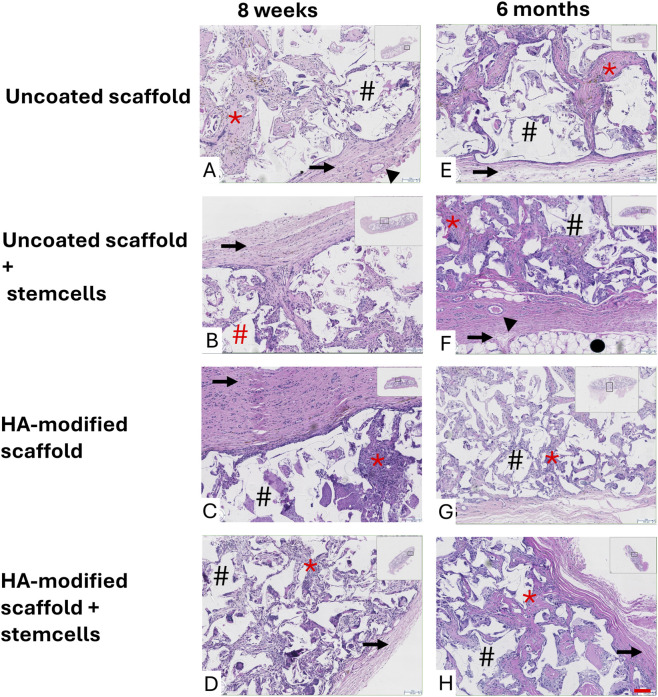
Histological evaluation after 8 weeks and 6 months. Scaffolds were surrounded by a well-organized fibrous connective tissue capsule (→), with elements of adipose tissue (●) and blood vessels (▲). Whitin the porous structure of the scaffolds a loose connective tissue was observed (*). The absence of mineralized tissue despite fibrous encapsulation reflects a common challenge in ectopic models, yet the principle remains relevant for clinical translation. The scaffold polymer is lost during processing of the samples, creating irregular artefacts of seemingly empty voids (#). H&E 10x. Scale-bar = 100 µm (H).

Explanted scaffolds were evaluated through the reconstruction and analysis following µCT scanning. After correcting threshold levels to account for the scaffold material, tissue ingrowth could be assessed, and at the 8-week time point, µCT analysis revealed significantly denser tissue across all groups, consistent with matrix secretion preceding osteoid deposition or the presence of pre-mineralized matrix. By 6 months, a reduction in tissue density was observed (Figure 6B). This decline highlights the limitation of transient HA exposure but also emphasizes the importance of optimizing scaffold coatings for long-term stability if applied in clinical maxillofacial settings (Figure 7).

#### 3.10.3 Histology

Histological evaluation of explanted scaffolds at both 8 weeks and 6 months revealed the presence of fibrous connective tissue surrounding the implants, forming a well-organized capsule at the scaffold periphery. Within the porous structure of the scaffolds, loose connective tissue infiltration was observed. No evidence of mineralized tissue or bone-like structures was detected in any of the experimental groups at either time point, despite the increased tissue radiodensity observed by µCT at 8 weeks. These findings indicate that tissue ingrowth within the scaffolds primarily consisted of non-mineralized connective tissue under the conditions tested.

## 4 Discussion

A key challenge in tissue engineering is the development of biomaterials that closely replicate the extracellular environment of target tissues (Chen et al., 2024). This study aimed to enhance interactions between cells and scaffolds by modifying an otherwise inert biomaterial with HA, a natural ECM component, which was incorporated onto the scaffolds *via* immersion coating. While HA coating modulated early cell–material interactions, the results demonstrate limited long-term osteogenic effects under the conditions tested. This surface modification was associated with upregulation of HA-specific surface receptors, namely, CD44 and RHAMM. Additionally, elevated expression levels of the bone-specific marker osteopontin in human BMSC and osteocalcin in rat BMSC were observed. Although PLATMC exhibits favorable properties for scaffold fabrication, its inherently low bioactivity may be improved through surface functionalization (Nair and Laurencin, 2007; Fukushima, 2016). While the biological activity of HA has been extensively documented (Zhai et al., 2020; Zha et al., 2016; Casale et al., 2016), there is still limited knowledge regarding the potential benefits of surface modification of synthetic polymers. Specifically, to our knowledge, HA-based surface modification of PLATMC scaffolds has not been thoroughly explored.

Enhancing the wettability of synthetic polymers has been shown to improve cell spreading and adhesion (Bu et al., 2019). In the present study, immersion coating of PLATMC scaffolds with HA led to a concentration-dependent increase in surface wettability, as demonstrated by contact angle measurements. HA contains numerous hydrophilic hydroxyl and carboxyl functional groups (Necas et al., 2008; Wang et al., 2003), which mimic natural ECM features and facilitate cell interaction (Laurent, 1987). By increasing the coating concentration, the number of these moieties likely increased proportionally across the scaffold surface, explaining the enhanced surface wettability observed. This may support improved cell seeding by enabling more uniform distribution of the cell suspension and facilitating greater initial contact with the scaffold. In BTE applications, scaffold wettability plays a key role in regulating cell adhesion, proliferation, and osteogenic differentiation (Hao et al., 2016). Importantly, these improvements in surface energy and receptor-mediated cell adhesion may prove relevant to dental tissue engineering, where scaffold–cell interactions determine the success of regenerative procedures. These effects are mediated by multiple mechanisms; The scaffold’s physicochemical properties can influence protein adsorption patterns, indirectly modulating cell responses, or directly affect cells through interactions with surface receptors (Xing Z. et al., 2020; Xing F. et al., 2020).

Pore size, interconnectivity, and overall porosity are critical scaffold parameters that impact cellular infiltration and tissue integration (Murphy et al., 2013; Tang et al., 2016; Yang et al., 2001). Porous scaffold designs increase the available surface area for cellular attachment and can be readily adjusted by selecting appropriate particle sizes and polymer volumes using salt leaching techniques as performed in the present study (Hou et al., 2003; Sola et al., 2019; Gremare et al., 2018), thereby facilitating reproducibility of scaffold parameters (Kwon et al., 2020). Excessively large pores, however, can impair cell–cell communication (Bruzauskaite et al., 2016), and cellular bridging (Murphy et al., 2013). Porosity also affects scaffold degradation and mass transport (Vert et al., 1992; Göpferich, 1996). Over time, tissue ingrowth reduces pore size (Yang et al., 2001), thus impacting nutrient exchange and waste removal (Murphy et al., 2013; Bruzauskaite et al., 2016). µCT analyses showed similar pore diameters (358–420 µm) across all scaffold groups, including uncoated controls, which fall within the range reported to support bone formation (Yang et al., 2001). However, optimal pore sizes vary depending on material and application (Murphy et al., 2013; Asadi et al., 2020), and indeed cell adhesion and migration are supported by different pore sizes (Bruzauskaite et al., 2016). All groups exhibited porosity greater than 80%, and HA treatment did not affect pore structure. Additionally, salt-leaching produced a rough surface, which is favorable for osteoblast adhesion (Dowling et al., 2011). µCT confirmed that HA coating had no detrimental effect on total surface area, porosity, pore size or fractal dimension—an indicator of surface complexity (Bertoldi et al., 2011). From a biofabrication perspective, the salt-leaching technique used here provides reproducible scaffold porosity and roughness, features that can be adapted to patient-specific needs in dentistry. While advanced 3D printing and bioprinting approaches are gaining attention, simple and scalable methods such as immersion coating remain valuable for translational applications.

Sterilization is essential for the clinical translation of biomaterials (Velasco et al., 2015), and in the present study autoclaving resulted in a significant reduction in HA molar mass, consistent with reports of pronounced thermal degradation at elevated temperatures (Stern et al., 2007). While Mn decreased significantly following sterilization, Mw showed only a modest, non-significant reduction. As HA bioactivity is strongly molar-mass dependent (Garantziotis et al., 2019), with different size fractions reported to exert distinct cellular effects, including on cell motility (Abatangelo et al., 2020), thermal processing must be considered in relation to the intended biological function of the material (Fuoco et al., 2019; Snetkov et al., 2020). Accordingly, reporting HA molar mass after all processing steps is essential. In this study, all HA-coated scaffold groups were prepared using the same sterilized HA material, and molar-mass–dependent biological effects were therefore not specifically investigated. While reduced HA molar mass may influence CD44 and RHAMM-mediated signaling and bias cellular responses toward early signaling events, internal comparisons between experimental groups remain valid, and future studies should explore alternative, lower-temperature sterilization strategies to better preserve polymer integrity.

Previous reports have shown that HA promotes cell adhesion (Zhao et al., 2015; Lai and Tu, 2012). In the present study, seeding efficiency ranged from 40─48% across all scaffold groups, with no significant differences. Seeding efficiency, cell spatial distribution and viability depend on multiple factors, including cell source, seeding duration, volume, density, and the physical properties of the scaffold material (Chen et al., 2011). µCT characterization did not reveal significant changes in scaffold physical properties following HA coating, suggesting that HA coating did not impair cell seeding.

Viability assessments using fluorescent staining and DNA quantification showed comparable values across all groups after 24 h, with a slight trend toward increased viability at higher HA concentrations. Analyses disclosed an overall positive effect of incubation time, with a significant increase in cell numbers, consistent with previous reports (Yassin et al., 2015). Importantly, this increase was consistent across all groups, suggesting that HA coating did not adversely affect cell expansion and may have supported cell attachment. It should be noted that DNA quantification provides an estimate of total cell number rather than a direct measure of proliferation rate, as it reflects the balance between cell division, survival, and potential cell loss. Previous studies have reported reduced cell proliferation on HA-coated substrates in a concentration- and molar mass-dependent manner, with decreased adhesion observed at higher molar masses or lower HA concentrations (Zhao et al., 2015; Corradetti et al., 2017). In the present study, HA molar mass was consistent across all experimental groups, precluding direct evaluation of this parameter. Other reports have demonstrated increased osteoblast proliferation when HA is added directly to the culture medium (Huang et al., 2003; Zou et al., 2008); however, under the present experimental conditions, surface-bound HA did not result in significant differences in DNA content between groups. These findings suggest that HA coating primarily supports cell viability and retention, rather than directly enhancing proliferation, and that proliferative responses may depend on HA presentation, concentration and molar mass.

Gene expression analysis demonstrated significant upregulation of CD44 at 24 h in the 0.5% HA-coated group compared to controls, aligning with prior findings in murine BMSC cultured on HA-coated substrates (Corradetti et al., 2017). The observed upregulation of CD44 may translate to enhanced adhesion of BMSC to the scaffolds. The interaction between CD44 and HA has been shown to regulate osteoblast differentiation by promoting the expression of osteogenic markers such as osteopontin, osteocalcin, and type I collagen. Additionally, this interaction contributes to maintenance of extracellular matrix integrity in bone tissue, which is essential for preserving bone strength and flexibility (Srinivasan et al., 2025). These findings support the role of HA as a bioactive surface modifier that enhances early cell–scaffold interactions and osteogenic priming in BTE applications.

RHAMM, another ligand and receptor involved in hyaluronan-mediated motility (Turley, 1992), was significantly upregulated at 24 h for all HA-coated groups. While analysis showed an overall decline in RHAMM expression by day 5, the 0.5% HA group consistently exhibited higher expression levels than controls at both timepoints. As both CD44 and RHAMM participate in key cellular processes related to scaffold-cell interactions (Bayer, 2020), their concurrent upregulation supports cell infiltration and adhesion during culture. This is particularly relevant because cell adhesion is an initial step in osteointegration, enabling cells to secrete ECM, and because these receptors mediate osteoblast attachment, where stronger adhesion may further support osteogenic differentiation. The co-upregulation of CD44 and RHAMM suggests a coordinated cellular response to HA-coated scaffolds, supporting both adhesion and migration. While CD44 facilitates stable attachment and cell–matrix interactions, RHAMM promotes cytoskeletal reorganization and directed cell movement (Srinivasan et al., 2025). Together, their expression indicates enhanced cell–biomaterial interaction, potentially contributing to not only improved cell survival and colonization and possibly early osteogenic signalling.

The release experiments demonstrated a rapid loss of HA from the scaffold surface, with HA becoming undetectable after a few days under *in vitro* conditions. This temporal profile indicates that the biological influence of HA in the present system is expected to be strongest during the early phases of cell–material interaction. Consequently, later cellular and tissue-level outcomes should not be attributed to sustained HA bioactivity, but rather to intrinsic properties of the PLATMC scaffold and to early HA-mediated priming effects. This distinction is particularly important when interpreting long-term *in vitro* and *in vivo* results, where HA is no longer present on the scaffold surface. Although modest trends in osteogenic marker expression were observed, these did not translate into statistically significant differences or increased mineral deposition. Collagen type I, a major component of osteoid and mature bone ECM (Forlino and Marini, 2016), showed a general trend of decline over time for all scaffold groups, potentially reflecting reduced ECM synthesis as cells reached confluence. RUNX2, a transcription factor essential for osteoblastic differentiation (Otto et al., 1997; Komori, 2010; Komori, 2002) and osteocalcin, a bone matrix protein expressed solely by mature osteoblasts (Lian et al., 1989) was detected at low, but similar levels across the groups. Notably, osteopontin, a bone ECM protein and marker associated with bone ECM remodeling (Singh et al., 2018), showed increased expression in the 0.5% HA-coated group. Although not statistically significant, this trend aligns with the hypothesized benefits of HA surface modification, namely, improved osteogenic differentiation and matrix production. These findings suggest that HA functionalization may enhance the bioactivity of PLATMC scaffolds, potentially promoting early osteogenic commitment in BMSC and fostering a more supportive environment for bone formation. Importantly, these trends warrant further investigation to clarify the specific contributions of HA coating to osteogenic differentiation and long-term bone regeneration outcomes.

Mineral deposition after 21 days was comparable across all scaffold groups, aligning with previous reports using rabbit BMSC (Zhao et al., 2015), which reported a positive correlation between the production of calcium deposition and the molar mass of HA higher than that employed here. Moreover, in the present study HA was released into the medium with residual HA levels decreasing after the first change of medium after 24 h and was undetectable after the second medium change on day 4. Although continuous replenishment of HA in cell culture medium has been shown to enhance mineralization *in vitro* (Zou et al., 2008), post-surgical modification of an implanted construct is not feasible and was therefore not incorporated in the present experiments. Consequently, the brief interaction between cells and the HA coating, prior to its release, may only have provided an initial osteogenic stimulus. Given the rapid release of HA, any biological effects attributable to the coating are necessarily confined to early time points, and long-term outcomes are primarily governed by the intrinsic properties of the PLATMC scaffold.

Evaluating the interaction between BMSC and HA-coated PLATMC scaffolds requires more than basic *in vitro* testing; it necessitates a robust preclinical model aligned with the research objectives. Accordingly, this study assessed the osteogenic potential of HA-modified scaffolds both *in vitro* and in an ectopic rodent subcutaneous implantation model. While the ectopic model is well suited for evaluating intrinsic material-driven osteoinductive potential, it does not replicate the biological and mechanical environment of craniofacial bone regeneration.

*In vivo* evaluations are essential for elucidating osteogenic potential of biomaterials, as *in vitro* assessments alone are insufficient to predict preclinical outcomes (Hatt et al., 2023). Based on the observed *in vitro* upregulation of CD44 and RHAMM and trends toward increased osteopontin expression, HA-modified scaffolds were evaluated *in vivo* using rBMSC-seeded constructs. Despite increased tissue density 8 weeks, ectopic mineralization could not be verified. Neither pristine, nor HA-coated PLATMC scaffolds demonstrated osteoinductive properties in the ectopic model, and this absence persisted at 6 months.

Consistent with the *in vitro* findings, HA leaching likely limited prolonged osteogenic stimuation *in vivo*. This effect may have been further constrained by the density of implanted cells. Poor vascularization, inherent to the subcutaneous model, likely compromised the survival of osteogenically committed cells and hindered subsequent bone formation. The limited HA retention on PLATMC, combined with the absence of endogenous osteoprogenitor cells and signaling molecules in the ectopic environment, supports the conclusion that PLATMC is not intrinsically osteoinductive and transient HA exposure is insufficient to overcome this limitation. These results align with previous reports demonstrating that PLATMC scaffolds support osteoconduction in orthotopic critical-sized defects (Hassan et al., 2022) but do not induce bone formation in ectopic models. Overall, the present findings reinforce that PLATMC scaffolds exhibit osteoconductive, but not osteoinductive, properties. For clinical translation in dentistry, strategies to prolong HA retention and enhance long-term osteoinductive potential will be critical, particularly in oral environments where scaffolds are exposed to mechanical loading.

Collectively, the results of the present study support our previous claim that the effect of HA on BMSC depends not only on size and concentration but also on the timing and duration of exposure. Future studies should focus on extending HA retention, refining HA-modification strategies, and evaluating these approaches in craniofacial and dental defect models to bridge the gap between experimental scaffolds and clinical applications.

## 5 Concluding remarks

In conclusion, surface modification of PLATMC scaffolds with hyaluronic acid *via* immersion coating improved surface wettability and supported early cellular engagement, as reflected by increased CD44 and RHAMM expression and osteogenic marker trends *in vitro*. Given the transient retention of HA, these effects are primarily relevant during initial cell–material interactions, while long-term outcomes appear to be governed by intrinsic scaffold properties and model-specific constraints. Together, the findings highlight the potential of HA-functionalized PLATMC scaffolds to modulate early cell adhesion and osteogenic signaling, while underscoring the need for strategies that improve HA retention to achieve sustained bioactivity in clinically relevant bone regeneration settings.

## 6 Limitations

A limitation of this study is the use of a subcutaneous ectopic implantation model without a barrier membrane. In clinical guided bone regeneration, membranes are routinely applied to exclude soft tissue and maintain a protected osteogenic space; their absence in the present model likely contributed to fibrous tissue infiltration and limited mineralization. However, the ectopic model was intentionally selected to evaluate the intrinsic osteoinductive potential of the scaffold under stringent conditions, independent of native bone cues or space-maintaining strategies.

An additional limitation relates to the transient retention of hyaluronic acid on the scaffold surface and the reduction in HA molar mass following sterilization. Although these factors do not compromise internal comparisons in the present study, they may restrict sustained bioactivity and long-term osteogenic effects. Accordingly, future studies should investigate strategies to improve HA retention and consider alternative sterilization approaches that better preserve polymer integrity.

Finally, evaluation of osteogenic potential of biomaterial scaffolds in orthotopic defect models will be necessary to more closely reflect clinically relevant regenerative conditions.

## Data Availability

The raw data supporting the conclusion of this article will be made available by the authors, without undue reservation.

## Supplementary material

Supplementary Figure S1 rBMSC adhesion and *in vitro* osteocalsin expression. Prior to implantation, rat BMSC were evaluated using confocal imaging. **(A)** Initial attachment of cells to the scaffolds. **(B)** At day 7, osteocalcin was evident in HA-coated scaffolds, not in the uncoated control group. (Scale-bar 100 μm). **(C)** Cell proliferation was assessed *in vitro via* DNA quantification, revealing a time-dependent increase in rBMSC numbers (*p* < 0.05), following a similar pattern to hBMSC (Figure 5A). **(D)** Mineralization at 21 days was similar between groups (Scale-bar 1 mm). Data are presented as mean ± SD, *n* = 5.
