# Supplementary figures and images for: Hyaluronic acid-coated Poly(L-lactide-co-1,3-trimethylene carbonate) modulate early cellular-scaffold interactions and osteogenic potential: a comprehensive in vitro and in vivo evaluation using mesenchymal stromal cells
Source: Front Bioeng Biotechnol. 2026 Jan 27;14:1740154. doi: 10.3389/fbioe.2026.1740154 (PMC12888217; doi:10.3389/fbioe.2026.1740154)

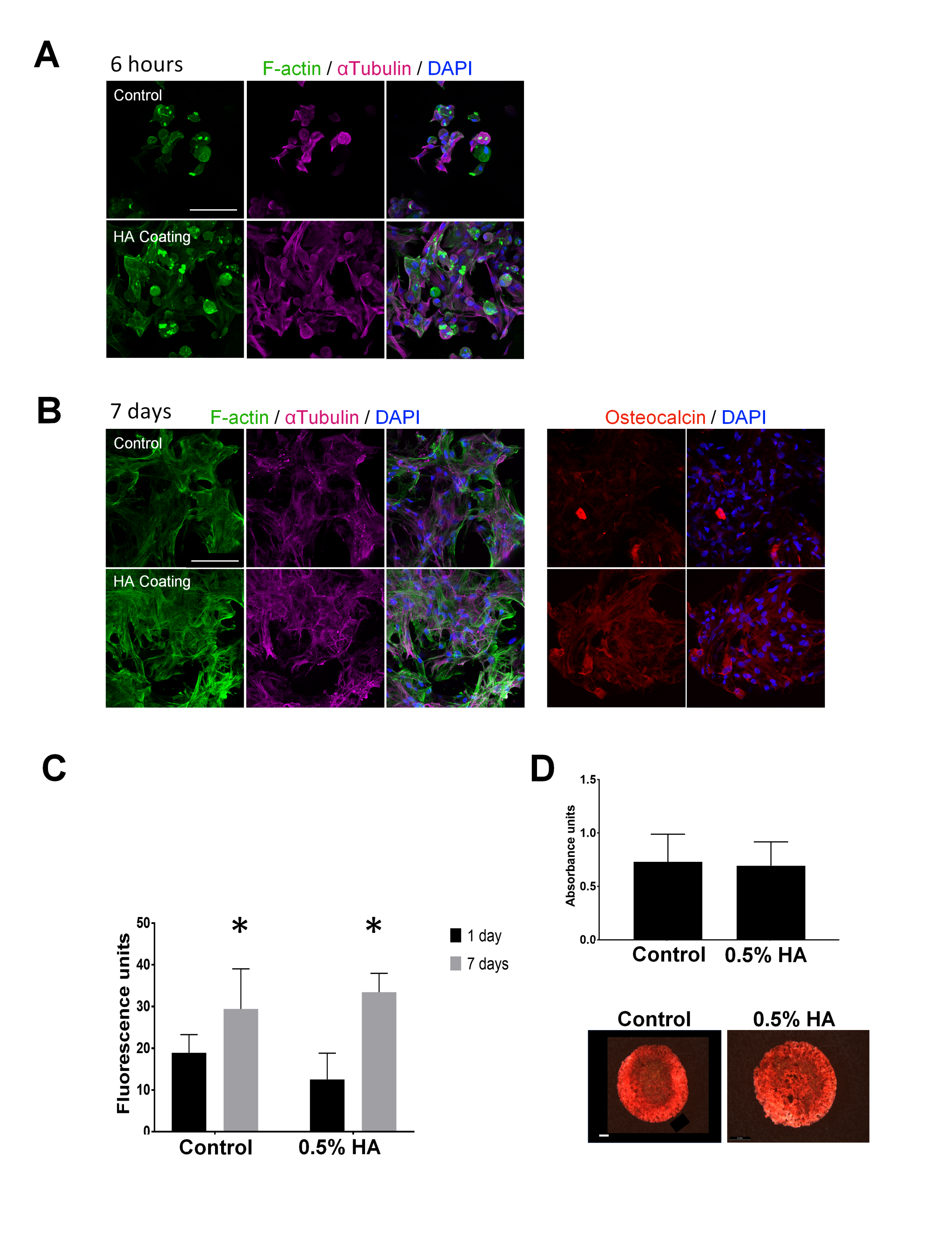

Supplement: Supplementary file 1 [file Image1.tif]
